# Efficient Degradation of Methylene Blue in Industrial Wastewater and High Cycling Stability of Nano ZnO

**DOI:** 10.3390/molecules29235584

**Published:** 2024-11-26

**Authors:** Ping Liang, Weiye Yang, Hongyan Peng, Shihua Zhao

**Affiliations:** 1 College of Physics and Electronic Engineering, Hainan Normol University, Haikou 571158, China; 202212070200007@hainnu.edu.cn (P.L.); haha_weiye@163.com (W.Y.); mdjphy@163.com (H.P.); 2 The Innovation Platform for Academicians of Hainan Province, Haikou 570228, China

**Keywords:** zinc oxide, degradation, cycling stability, photocatalysis, methylene blue (MB)

## Abstract

Photocatalytic degradation research has attracted significant attention in the wake of the increasingly severe global challenge of water pollution. In this study, nano-ZnO was synthesized through a straightforward method using zinc acetate anhydrous as the precursor. The experimental results revealed that annealing conditions significantly influenced the bandgap energy (Eg) of ZnO, with a positive correlation observed between the intensity of photoluminescence (PL) spectra and photocatalytic activity. Under optimal annealing conditions at 300 °C for 1.5 h, the photocatalytic degradation efficiency of methylene blue (MB) exceeded 98% within 40 min of ultraviolet (UV) irradiation, surpassing the efficiencies reported for ZnO in recent studies. This high performance underscores the excellent photocatalytic activity of the synthesized ZnO samples. Furthermore, after five photocatalytic cycles, the degradation efficiency of MB remained above 90%, and the crystalline structure of the ZnO samples remained stable, demonstrating their exceptional structural stability during the photocatalytic process. Additionally, this study examined the effects of stirring conditions and different light sources on MB degradation, providing valuable insights for future research in related fields.

## 1. Introduction

Water pollution is increasingly recognized as a critical global challenge requiring immediate attention [1]. The discharge of industrial wastewater and domestic sewage introduces significant quantities of harmful substances, including carcinogenic chemicals, into aquatic systems. These pollutants not only cause severe ecological damage but also pose serious risks to human health [2], highlighting the urgent need for effective wastewater treatment.

As a significant sector of chemical manufacturing, the dye industry supplies a wide range of products to industries such as textiles, printing, food, and pharmaceuticals. However, it also introduces various environmental and health challenges. Wastewater generated during dye production contains high levels of organic matter, heavy metals, residual dyes, and other hazardous compounds. If discharged untreated, it can severely pollute rivers and lakes, disrupt aquatic ecosystems, and compromise water quality [3]. Organic substances from dye wastewater or wastewater used for agricultural irrigation can accumulate in soil, degrading its quality, impairing crop growth, and even rendering the land infertile [4]. The emissions of volatile organic compounds (VOCs), nitrogen oxides, and sulfur oxides during dye manufacturing contribute to air pollution, exacerbate the greenhouse effect and acid rain, and deteriorate air quality [5]. Harmful chemicals in dyes, including aromatic amines, heavy metals (e.g., lead, cadmium, and chromium), and azo dyes, can bioaccumulate through the food chain, with prolonged exposure linked to serious health issues such as cancer, reproductive disorders, and neurological damage [6]. Water and soil contamination reduces the ecological carrying capacity, causing the death of many aquatic plants and animals unable to adapt to degraded habitats, thereby reducing biodiversity [7].

Methylene blue, a member of the triphenylmethane dye family, exhibits a unique hydrophilic-lipophilic balance, enabling stable bonding to various fiber surfaces. It demonstrates particularly effective coloring performance on natural fibers such as cotton, linen, and viscose [8]. In traditional dyeing processes, methylene blue functions as a direct dye, imparting fabrics with a blue hue characterized by depth and elegance [9]. However, the textile dyeing industry represents a significant global source of pollution, as the discharge of untreated dye wastewater not only depletes valuable water resources but also introduces organic pollutants that pose serious threats to ecosystems and human health [10]. Methylene blue is no exception. Its production and application can result in persistent residues that are difficult to degrade, posing potential risks to aquatic ecosystems [11].

ZnO, a widely used non-toxic photocatalyst, exhibits significant potential in photocatalysis. Due to its relatively wide bandgap [12,13], ZnO can absorb visible light, enabling operation under natural sunlight and reducing reliance on UV light sources. It demonstrates excellent chemical stability during photocatalytic processes, facilitating reuse and lowering long-term operational costs. Furthermore, ZnO is environmentally and biologically safe, making it a promising candidate for applications in wastewater treatment [14], air purification [15], and other environmental fields [16,17].

Researchers at Gustave Eiffel University have investigated methods to improve ZnO photocatalysts for water purification. Their studies focused on various synthesis techniques and structural modifications to enhance photocatalytic performance. The research aims to utilize the photocatalytic properties of ZnO to degrade organic pollutants in water, thus mitigating environmental contamination [18]. The team led by Abayomi D. Folawewo and Muhammad D. Bala studied ZnO composites for treating dye-contaminated wastewater. Their work emphasized the development of heterostructure ZnO photocatalysts, achieving improved dye degradation under visible light, with the goal of addressing industrial wastewater challenges, particularly in the dyeing industry [19]. Similarly, the group led by Sharifah Bee Abdul Hamid at the University of Malaya focused on the mechanism of water oxidation on ZnO surfaces, aiming to enhance the efficiency of photoelectrochemical water-splitting devices. This research plays a significant role in developing renewable hydrogen production methods through water splitting [20]. However, many of these studies involve complex preparation procedures that are challenging to implement. In this study, a facile synthesis method was employed to prepare ZnO photocatalytic materials under varying annealing conditions. The photocatalytic degradation of MB under different light conditions was evaluated, and the catalytic cycling and stability were analyzed. Although the ZnO nanorods were prepared using a traditional hydrothermal method and the material dosages differed among various reports, the ZnO nanorods synthesized in this study exhibited a remarkable degradation effect on MB after only 40 min of light irradiation. While most studies report degradation efficiencies under longer light exposure, this research demonstrates that optimized material dosages can achieve superior degradation efficiency within a shorter timeframe.

## 2. Results and Discussion

### 2.1. XRD Analysis

**Figure 1:**
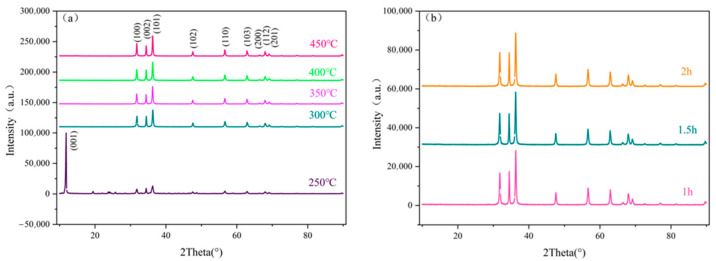
(**a**) XRD patterns of ZnO sample annealed for 2 h at different annealing temperatures; (**b**) XRD patterns of ZnO annealed at 300 °C for different annealing times.

Figure 1a presents the XRD patterns of ZnO samples annealed for 2 h at various temperatures, while Figure 1b shows the XRD patterns for samples annealed at 300 °C for different durations. A comparison with the standard card for hexagonal wurtzite ZnO reveals diffraction peaks corresponding to ZnO crystal planes, including (100), (002), (101), (102), (110), (103), (200), (112), and (201) [21,22,23]. In Figure 1a, the sample annealed at 250 °C for 2 h displayed a sharp peak at the (001) crystal plane of zinc acetate, indicating an incomplete reaction of the zinc acetate at this temperature. Part of the anhydrous zinc acetate was converted to ZnO, while some remained unreacted [24]. Additionally, the increasingly sharp peak at the (101) crystal plane in Figure 1a suggests that higher annealing temperatures lead to enhanced crystallinity [25,26]. In contrast, Figure 1b shows that the annealing time does not significantly affect the crystal phase structure of ZnO.

### 2.2. SEM and Mapping Analysis

**Figure 2:**
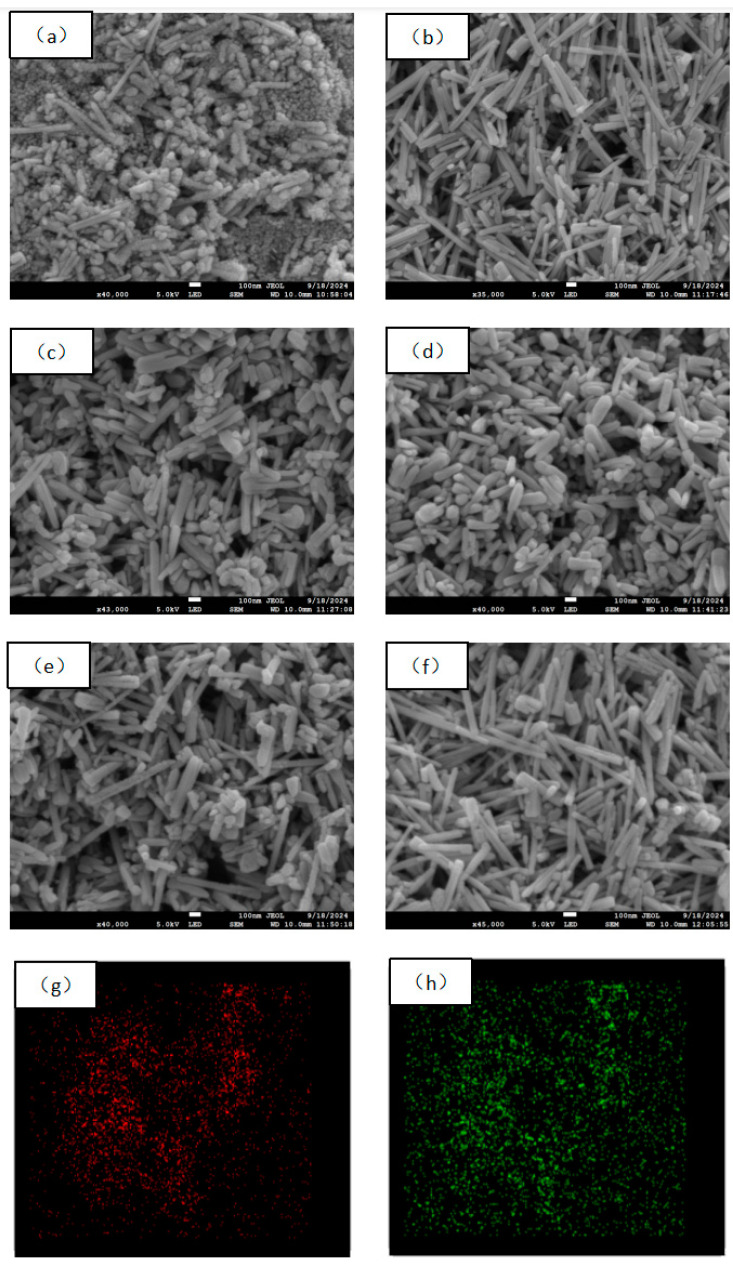
SEM images of ZnO samples prepared under different annealing conditions: (**a**) 250 °C for 2 h, (**b**) 300 °C for 2 h, (**c**) 350 °C for 2 h, (**d**) 400 °C for 2 h, (**e**) 300 °C for 1 h, and (**f**) 300 °C for 1.5 h. Mapping diagram of samples annealed at 300 °C for 1.5 h; (**g**) Zn element, (**h**) O element.

Figure 2a presents the SEM image of a ZnO sample annealed at 250 °C for 2 h. As shown in Figure 2a, the surface particles exhibit irregular shapes, with some forming rod-like structures and others appearing as particles. This behavior is attributed to the incomplete reaction of anhydrous zinc acetate at 250 °C. Figure 2b shows the ZnO sample annealed at 300 °C for 2 h. It can be observed that more uniform rod-like structures emerge, with a diameter of approximately 90 nm and a length of about 1 μm. At lower annealing temperatures, grain growth initiates but does not result in a fully uniform morphology. Figure 2c illustrates the ZnO sample annealed at 350 °C for 2 h. From Figure 2c, it is evident that the rod-like structures become shorter, and numerous small particles are present [27]. Figure 2d depicts the ZnO sample annealed at 400 °C for 2 h. The rod-like structures are notably shorter and thicker, with both ends gradually forming hemispherical shapes. Figure 2e shows the ZnO sample annealed at 300 °C for 1 h, exhibiting minimal morphological changes compared to Figure 2b, suggesting that the annealing time has a relatively minor effect on grain morphology. Figure 2f displays the ZnO sample annealed at 300 °C for 1.5 h, where the rod-like structures remain well-defined and more uniform compared to Figure 2b,e. While increasing the annealing time impacts morphology to some extent, its effect is weaker relative to that of the annealing temperature [28]. At the same annealing temperature, longer annealing times lead to more regular particle morphology and promote directional grain growth. Figure 2g,h show the elemental distribution maps of the ZnO sample annealed at 300 °C for 1.5 h. Figure 2g illustrates the Zn distribution in red, while Figure 2h shows the O distribution in green. The red spots represent the presence and distribution of zinc in the sample. The slightly dispersed pattern suggests a uniform distribution of zinc within the material. The green spots indicate the distribution of oxygen in the ZnO material [29]. Compared to zinc, the oxygen distribution appears more abundant, suggesting a more uniform and higher concentration of oxygen in the sample. This observation is consistent with the chemical structure of ZnO, where oxygen is typically more prevalent and plays a crucial role in photocatalytic performance.

### 2.3. FTIR Analysis

**Figure 3:**
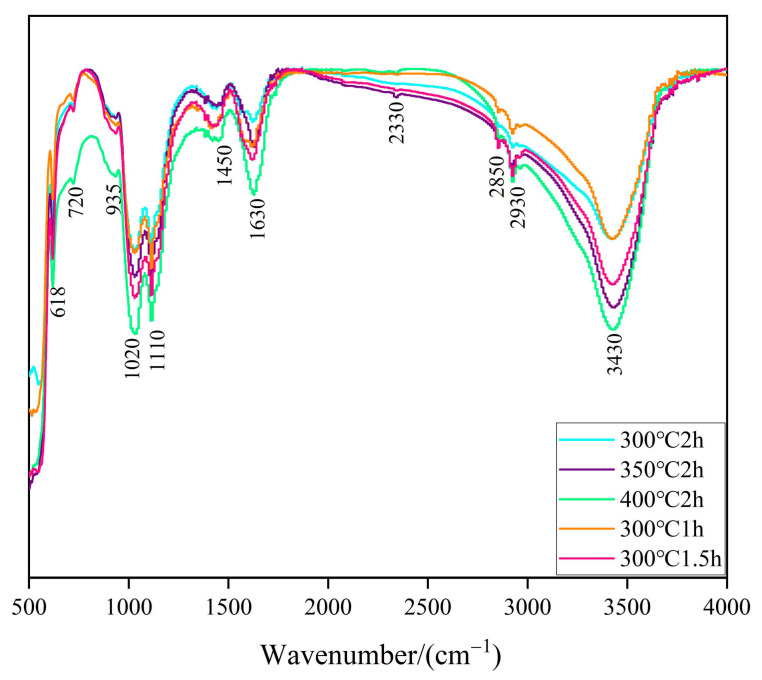
Infrared spectra of ZnO prepared under different annealing conditions.

Figure 3 presents the infrared spectra of zinc oxide prepared under various annealing conditions. The peaks at 618 cm^−1^ and 720 cm^−1^ are attributed to the bending vibrations of Zn-O bonds, indicating the presence of amorphous or defective ZnO, as well as different types of defects or amorphous regions within the material [30]. The peaks at 935 cm^−1^ and 1020 cm^−1^ correspond to the O-H stretching vibrations of water molecules adsorbed on the surface, suggesting the presence of H_2_O on the sample surface. The peak at 1110 cm^−1^ is related to the symmetric or asymmetric stretching vibrations of the Zn-O bond, particularly in polycrystalline or amorphous ZnO [31]. The strength and stability of the Zn-O bonds are closely linked to photocatalytic activity, as they influence the mobility and reactivity of photogenerated electrons and holes on the ZnO surface. The peaks at 1450 cm^−1^ and 2330 cm^−1^ are associated with the C=O stretching vibrations of CO_2_, indicating the presence of adsorbed carbon dioxide on the sample. The peak at 1630 cm^−1^ corresponds to the bending vibration of the H-O-H bond. The peaks at 2850 cm^−1^ and 2930 cm^−1^ are related to the C-H stretching vibrations of alkyl groups, corresponding to the symmetric and asymmetric stretching absorption peaks of CH_2_. The peak at 3430 cm^−1^ is associated with the O-H stretching vibrations in H_2_O molecules [32]. In photocatalytic reactions, H_2_O molecules can act as electron donors or acceptors, participating in the transfer processes of photogenerated electrons and holes, thereby facilitating photocatalytic water splitting.

### 2.4. Light Absorption Performance and Bandgap Analysis

**Figure 4:**
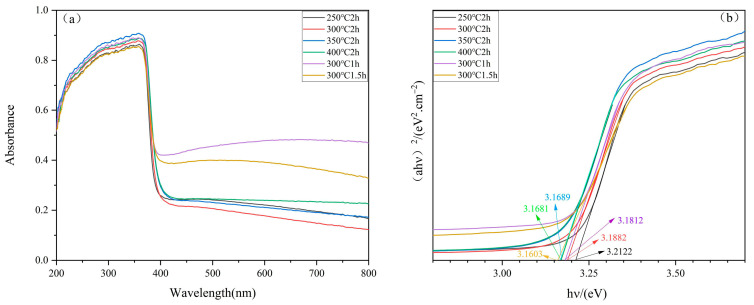
(**a**) UV-Vis diffuse reflectance spectra of ZnO under different annealing conditions; (**b**) (αhν)^2^−hν curve of different annealing conditions.

Figure 4a presents the UV-Vis spectra of ZnO under different annealing conditions. The ZnO nanostructures exhibit a broad absorption band in the ultraviolet region, with a peak at 365 nm. This strong absorption is attributed to the electronic transition from the valence band to the conduction band, reflecting the semiconductor nature of ZnO. Additionally, the peak corresponds to the electronic transition from O^2−^ to Zn^2^⁺, providing direct evidence of the material’s optical activity [33]. The Tauc method is employed to analyze the relationship between the absorption coefficient and photon energy, which enables the precise calculation of the Eg of ZnO.

(1) $${\left(\alpha h\nu \right)}^{2}=A(h\nu -Eg)$$

where α represents the intrinsic absorption coefficient, hν denotes the photon energy, and A represents the transition constant.

Figure 4b shows the relationship between (αhν)^2^ and hν for ZnO samples under different annealing conditions [34]. As shown in Figure 4b, the annealing conditions have a significant impact on the Eg values, as summarized in Table 1. The ZnO sample annealed at 300 °C for 1.5 h exhibits an Eg value of 3.1603 eV, which is lower than the sample annealed for 2 h at the same temperature [35]. ZnO is a typical wide-bandgap semiconductor, with an intrinsic bandgap of approximately 3.37 eV (368 nm) [36]. This indicates that, in their natural state, electrons require at least 3.37 eV of energy to transition from the valence band to the conduction band. However, the prepared ZnO sample has a bandgap lower than 3.37 eV, which reduces the energy needed for electron transitions, making it easier to absorb photons across a broader frequency range and resulting in a greater generation of electron-hole pairs [37]. These findings not only underscore the influence of annealing conditions on the optical properties of ZnO nanostructures but also provide a theoretical basis for their use in photocatalysis and photoconversion, highlighting the potential of ZnO in energy and environmental applications [38,39].

### 2.5. XPS Analysis

**Figure 5:**
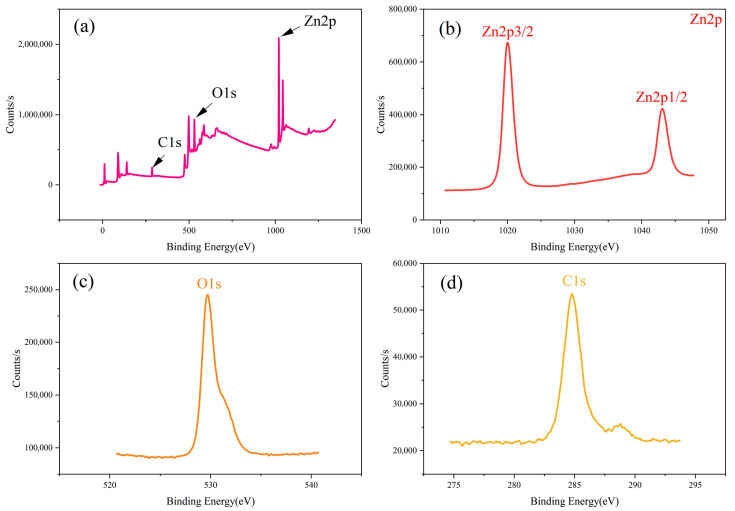
(**a**) XPS full spectrum of ZnO annealed at 300 °C for 1.5 h; (**b**) Zn2p; (**c**) O1s; (**d**) C1s.

To investigate the valence states of the ZnO material, XPS analysis was performed on one of the samples. Figure 5 presents the XPS spectra of the ZnO sample annealed at 300 °C for 1.5 h. As shown in Figure 5a, the full XPS scan from 1 to 1400 eV reveals the presence of three elements: Zn2p, O1s, and C1s. Figure 5b shows the Zn2p1/2 and Zn2p3/2 peaks, which typically form a pair of symmetric peaks due to the spin-orbit splitting effect of electrons in the 2p orbital. The peaks at 1020.1 eV and 1043.2 eV correspond to Zn2p3/2 and Zn2p1/2, respectively. The energy difference between these peaks is 23.1 eV, which is consistent with the standard value of 22.97 eV, indicating that Zn ions are in the +2 oxidation state in the oxide. This is fundamental to the stable structure of ZnO [40]. The hexagonal wurtzite structure of ZnO depends on the charge balance between Zn^2^⁺ and O^2−^, which is essential for the efficient photocatalytic properties of the material. Figure 5c shows the O1s peak centered at 529.7 eV, which is characteristic of oxide bonds formed between oxygen atoms and metal ions [41,42]. This feature significantly influences the chemical properties of the ZnO surface. During photocatalysis, the surface oxide bonds play a key role in the generation, separation, and interaction of photogenerated electrons and holes with reactant molecules. Figure 5d shows the presence of carbon, which is attributed to the instrument.

### 2.6. PL Analysis

**Figure 6:**
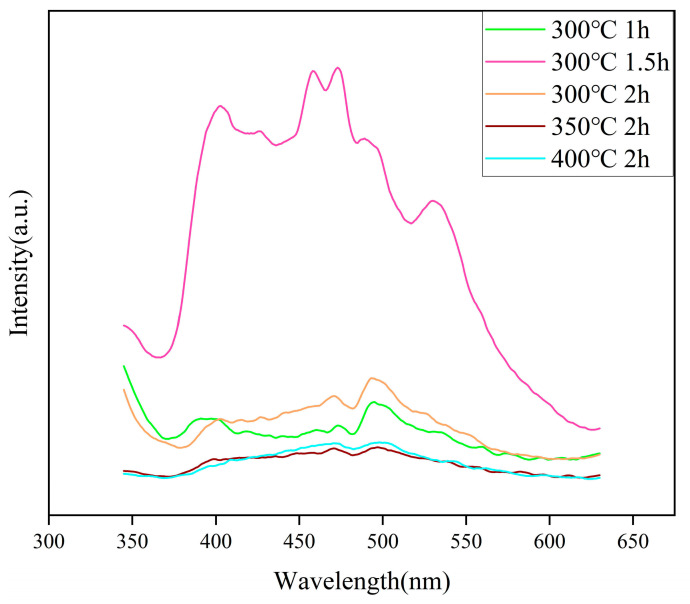
PL spectra of ZnO under different annealing conditions.

Figure 6 presents the PL spectra of the prepared ZnO samples. The excitation wavelength used is 325 nm, and the PL peak at 401 nm, shown in Figure 6, is attributed to the emission at the edge of the wide bandgap of ZnO, resulting from free exciton recombination. The blue emission in the 410–440 nm range is ascribed to the recombination of electrons in the conduction band with holes in oxygen vacancies, or the recombination of holes in the valence band with interstitial zinc defects. The green emission at 534 nm originates from the recombination of electrons from ionized oxygen vacancies on the surface with photoexcited holes in the valence band. In addition to these emission peaks, additional peaks at 453, 469, 482, and 492 nm are observed, corresponding to blue-green emissions [43].

It should be noted that Figure 6 demonstrates a positive correlation between the PL spectrum intensity and photocatalytic activity, which is particularly evident in the ZnO sample annealed at 300 °C for 1.5 h. This observation suggests a potential mechanism for the enhanced photocatalytic activity, in which oxygen vacancies play a crucial role. The presence of surface oxygen vacancies effectively traps photogenerated electrons, facilitating the formation of free or bound excitons, thereby increasing the PL intensity. A higher concentration of oxygen vacancies correlates with an increase in PL spectrum intensity [44]. As a defect state, oxygen vacancies provide additional energy levels and act as temporary “storage” for electrons or holes, preventing their rapid recombination [45]. Once captured, these carriers can be released under suitable conditions and contribute to subsequent photocatalytic reactions, rather than being immediately recombined and consumed [46]. This increases the number of available charge carriers, and these excitons serve as efficient sources of photocatalytic activity [47]. Furthermore, oxygen vacancies modify the surface electronic structure of ZnO, enhancing surface active sites, providing more favorable conditions for photocatalytic reactions, and promoting the adsorption of reactants and the dissociation of products [48].

### 2.7. ZnO Photodegradable MB Performance

**Figure 7:**
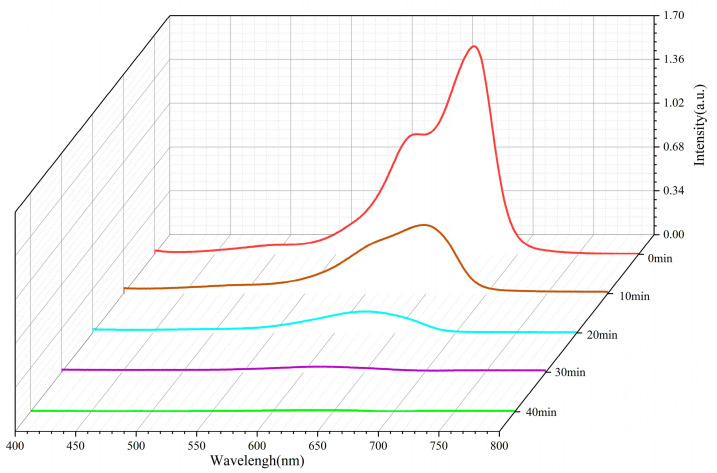
UV-Vis absorption spectra of ZnO samples annealed at 300 °C for 1.5 h.

Figure 7 presents the UV-Vis absorption spectrum of the ZnO sample annealed at 300 °C for 1.5 h. The zinc oxide was synthesized by annealing anhydrous zinc acetate at 300 °C for 1.5 h and subsequently placed in 50 mL of 10 mg/L MB solution. Every 10 min, 3 mL of the solution was extracted, and the pollutant was gradually degraded. The characteristic absorption peak of methylene blue in the UV-Vis spectrum typically appears around 665 nm. The intensity of this peak is proportional to the concentration of MB [49,50]. Prior to the photocatalytic reaction, a clear and intense absorption peak is observed. As the photocatalytic reaction progresses, MB is degraded, leading to a decrease in its concentration and a corresponding reduction in the absorption peak intensity at 665 nm. By comparing the peak intensities at various time points, the degradation efficiency of MB can be evaluated [51,52].

**Figure 8:**
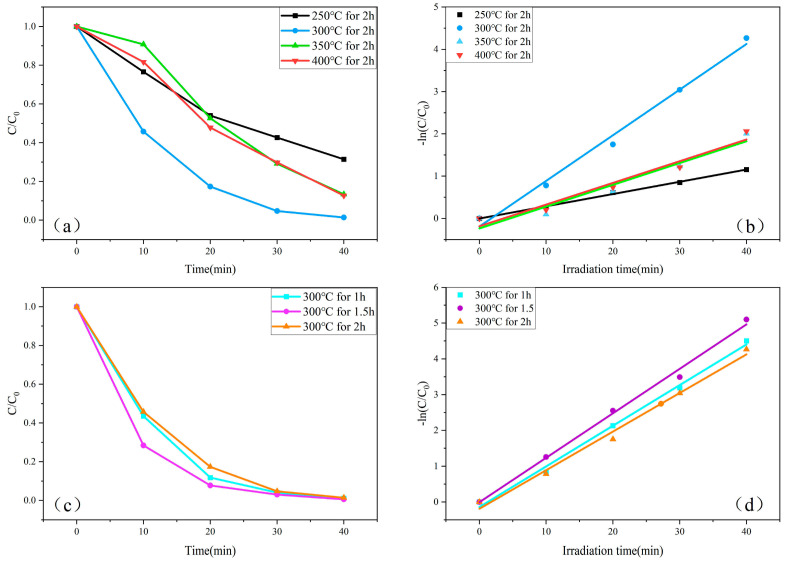
(**a**) Comparison of photodegradation of MB under UV irradiation at different annealing temperatures. (**b**) Reaction kinetics of ZnO samples at different annealing temperatures. (**c**) Comparison of photodegradation of MB under UV irradiation for different annealing time. (**d**) Reaction kinetics of ZnO samples for different annealing time.

Figure 8a compares the photocatalytic degradation of MB under UV light irradiation by ZnO samples annealed at different temperatures. From Figure 8a, it can be observed that the degradation efficiency of MB by the ZnO samples annealed for 2 h follows the trend: η_300 °C_ > η_250 °C_ > η_400 °C_ > η_350 °C_. This phenomenon can be explained by the fact that, within a certain range, an optimal annealing temperature enhances the crystallinity and grain size of ZnO, thereby improving its photocatalytic activity. At 300 °C, ZnO exhibits the optimal crystalline structure and grain size, which facilitates the separation of photogenerated electron-hole pairs, reduces recombination, and enhances photocatalytic efficiency [53]. However, when the annealing temperature is further increased to 400 °C, grain growth is promoted, leading to larger grain sizes. This increases the migration distance of photogenerated carriers, which hinders electron-hole separation, thus reducing photocatalytic efficiency. At 250 °C, incomplete decomposition of zinc acetate, as indicated by XRD results, results in poor ZnO crystallinity with numerous defects, which also impairs photocatalytic performance [54]. Figure 8b shows the relationship between $-\ln(\frac{C}{C_{0}})$ and UV irradiation time (t) at different annealing temperatures. The linear regression fit produces a straight line, indicating that the photocatalytic degradation of MB by the synthesized ZnO samples follows first-order reaction kinetics.

$$-\ln\left(\frac{C}{C_{0}}\right)=k_{a}t$$

The apparent rate constant ka represents the reaction rate, while C_0_ and C denote the initial concentration of MB solution at adsorption–desorption equilibrium and the remaining concentration of MB during the photocatalytic reaction, respectively. In the linear fitting of kinetic curves, R^2^ is a coefficient that measures the difference between a data point and its predicted value from the linear regression model. The value of R^2^ ranges from 0 to 1, and the closer it is to 1, the better the curve fit. As shown in Figure 8b, the ZnO sample annealed at 300 °C for 2 h exhibits the fastest degradation rate. Figure 8c presents the photocatalytic degradation of MB under UV light by ZnO samples annealed at 300 °C for different annealing times. As indicated in Figure 8c, the degradation efficiency follows the order: η_1.5 h_ > η_1 h_ > η_2 h_, suggesting that annealing time affects the structural perfection and defect states of ZnO. The annealing time of 1.5 h allows ZnO to achieve an optimal balance between structural refinement and defect states, resulting in the highest photocatalytic efficiency. An annealing time of 1 h is insufficient for complete structural optimization, while 2 h of annealing leads to excessive heat treatment, increasing grain size, eliminating defects, and reducing the efficiency of photogenerated carrier separation [55]. By adjusting the annealing temperature and time, the structure and defect states of ZnO can be optimized, influencing the separation and migration of photogenerated carriers, ultimately determining the photocatalytic efficiency. Appropriate annealing conditions can optimize these parameters, enhancing the degradation efficiency of pollutants such as methylene blue [56]. Figure 8d illustrates the relationship between $-\ln(\frac{C}{C_{0}})$ and UV irradiation time (t), demonstrating the highest catalytic activity of the ZnO sample annealed at 300 °C for 1.5 h. Table 2 presents the degradation rates, first-order reaction rate constants ka, and R^2^ values for ZnO under various conditions. A comparison of the ka values in Table 2 further confirms that the sample annealed at 300 °C for 1.5 h achieves the highest catalytic efficiency.

**Figure 9:**
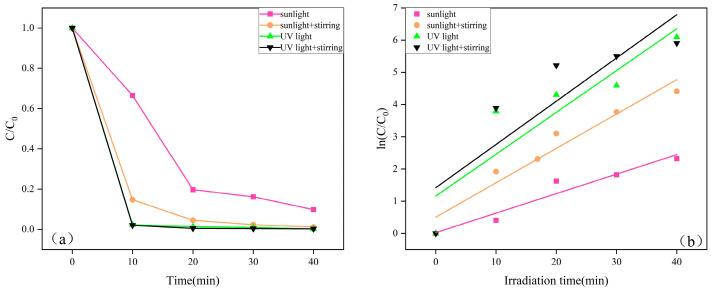
(**a**) The reflection of stirring and light conditions on the photocatalytic degradation of MB. (**b**) Reaction kinetics of a sample with different stirring and light conditions.

To further explore the effects of sunlight, ultraviolet (UV) light, and stirring on photocatalytic performance, four experimental conditions were tested: (1) sunlight without stirring, (2) sunlight with stirring, (3) UV light without stirring, and (4) UV light with stirring. By comparing the photocatalytic effects of ZnO under these conditions, the influence of external factors on photocatalytic efficiency was investigated. Figure 9a illustrates the photocatalytic degradation of MB under different light sources and stirring conditions. From Figure 9a, the photocatalytic efficiency under various conditions follows the order: η _(sunlight with stirring)_ > η_(sunlight)_ > η_(UV light with stirring)_ > η_(UV light)_. This outcome is attributed to the higher total light intensity of sunlight compared to the UV light source in the laboratory. Although the UV radiation proportion in sunlight is lower than that of the UV lamp, its broader spectral range, which includes parts of the visible light spectrum, enables ZnO to absorb additional wavelengths and enhance photocatalytic activity. Stirring promotes convective flow in the solution, increasing the reaction rate of reactants on the photocatalyst surface and facilitating mass transfer, thereby improving photocatalytic efficiency [57]. Additionally, Figure 9b shows the reaction kinetics for degradation under different light sources and stirring conditions. A comparison of the ka values in Table 2 supports this observation, although the effect varies under different lighting conditions. The experiments performed under sunlight were conducted in an open environment, which facilitates better interaction between oxygen in the air, other reactants, and the ZnO catalyst, thereby enhancing photocatalytic efficiency [58]. These findings provide an experimental basis for the industrial application of ZnO in photocatalytic MB degradation. Table 3 compares the efficiency of other studies in degrading MB. From Table 3, it is evident that the ZnO sample prepared by annealing at 300 °C for 1.5 h exhibits a strong photocatalytic effect and notable advantages in energy conservation and emission reduction.

### 2.8. Catalyst Recycling and Stability Testing

**Figure 10:**
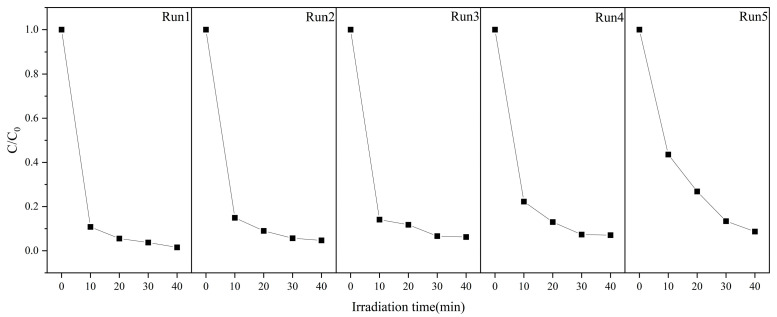
Cyclic experiment of photocatalytic degradation of MB.

**Figure 11:**
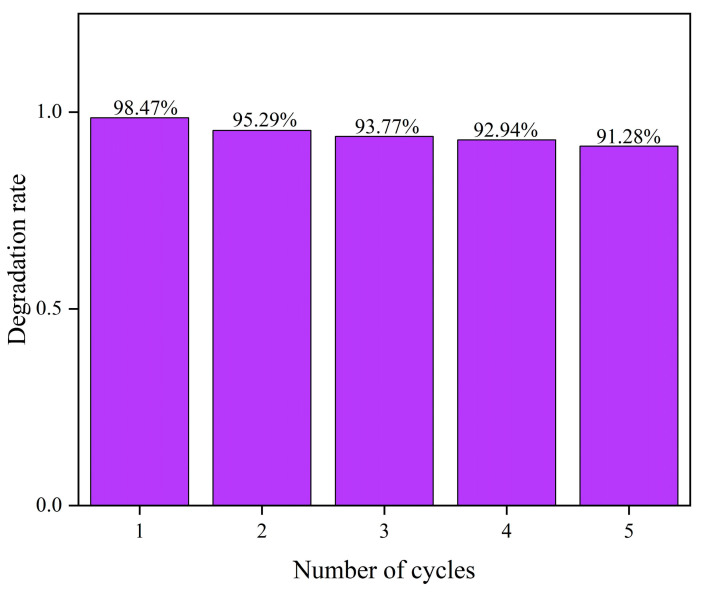
The catalytic efficiency of photocatalytic degradation of MB during cycles.

As shown in Figure 8, the ZnO sample annealed at 300 °C for 1.5 h exhibits a significant enhancement in photocatalytic degradation performance. This result highlights not only the high catalytic activity of the material but also its potential for stability and recyclability. Subsequently, catalyst recycling experiments were conducted to assess the stability of ZnO, providing a foundation for its industrial application. Figure 10 presents the cyclic photocatalytic degradation experiments of MB using the prepared ZnO as the catalyst. As seen in the figure, the catalytic efficiency in the fifth cycle reached 60% after 10 min and 90% after 40 min. Figure 11 shows the catalytic efficiency of ZnO annealed at 300 °C for 2 h across five cycles. In the photocatalytic recycling experiment, 250 mg of the ZnO catalyst (annealed at 300 °C for 1.5 h) was used to degrade 250 mL of 10 mg/L MB solution. As shown in Figure 11, the degradation rates for each cycle were 98.47%, 95.29%, 94.77%, 92.94%, and 91.28%, respectively. Although the degradation rate gradually decreased with each cycle, it remained above 90%, reflecting the stability and durability of the catalyst. The optimized annealing treatment imparts high structural and chemical stability to ZnO, indicating that even after multiple cycles, the active sites and structure of the catalyst remain nearly unchanged, maintaining high photocatalytic activity. Furthermore, ZnO catalysts prepared under optimized conditions exhibit excellent electron-hole pair separation efficiency, which is sustained even after multiple cycles. This ensures that photogenerated charge carriers effectively participate in the catalytic reaction, maintaining a high degradation rate [73]. The results underscore the importance of optimizing preparation conditions, such as annealing temperature and time, to enhance both catalyst performance and recycling stability.

### 2.9. SEM and XRD After Recycling

**Figure 12:**
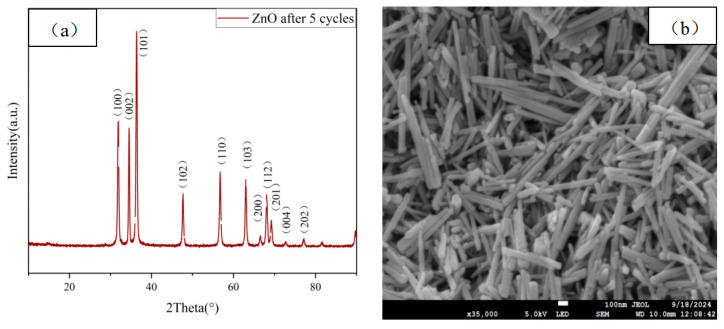
After 5 cycles of photocatalytic degradation of MBs, the ZnO samples were annealed at 300 °C for 1.5 h with (**a**) XRD and (**b**) SEM.

Figure 12a presents the XRD patterns of ZnO samples after five cycles of photocatalytic degradation of MB. As shown in Figure 12a, the diffraction peaks of ZnO after five cycles match those observed in Figure 1b. Figure 12b shows the SEM images of the ZnO samples after five cycles of photocatalytic degradation of MB, indicating that the morphology of ZnO closely resembles that of the freshly prepared samples. Both XRD and SEM analyses reveal no significant changes in the crystal structure of ZnO after multiple photocatalytic cycles, demonstrating that the ZnO catalyst exhibits excellent structural stability during the photocatalytic process [74].

### 2.10. ZnO Photocatalytic Mechanism Diagram

**Figure 13:**
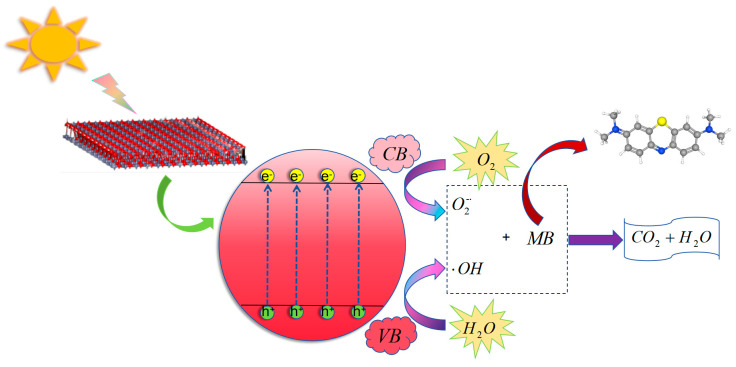
The schematic mechanism diagram of ZnO photocatalytic degradation of MB.

Figure 13 shows the schematic photocatalytic mechanism of ZnO. Upon excitation by light or thermal energy, zinc oxide generates electron (e^−^) and hole (h⁺) pairs. These reactive species participate in redox reactions, facilitating pollutant degradation. When zinc oxide absorbs light, electrons in the valence band are excited to the conduction band, leaving behind holes in the valence band, and resulting in the formation of photogenerated electron-hole pairs [75]. The electrons and holes can then migrate to the surface of ZnO, where they react with adsorbed oxygen and water molecules to produce superoxide anions (O_2_^−^) and hydroxyl radicals (OH), respectively. These reactive oxygen species exhibit strong oxidative properties, promoting the mineralization of organic pollutants into harmless inorganic substances, such as carbon dioxide and water [76].

## 3. Materials and Methods

### 3.1. Preparation of Zinc Oxide

**Figure 14:**
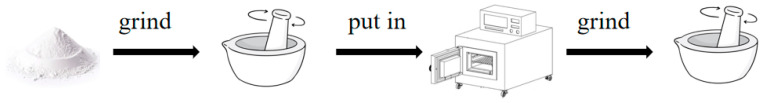
Schematic diagram of the experimental process for the preparation of zinc oxide.

Figure 14 is a schematic diagram of the experimental process for the preparation of zinc oxide. A certain amount of anhydrous zinc acetate was ground for 10 min and then annealed in a muffle furnace to form zinc oxide. The heating rate of the box type resistance furnace SX_2_-2.5-12 (Boyuntong Instrument Technology Co., Ltd., Nanjing, China)used in this experiment is 10 °C per min. The resulting product was subsequently ground for another 10 min.

### 3.2. Photocatalytic Degradation of Dyes

**Figure 15:**
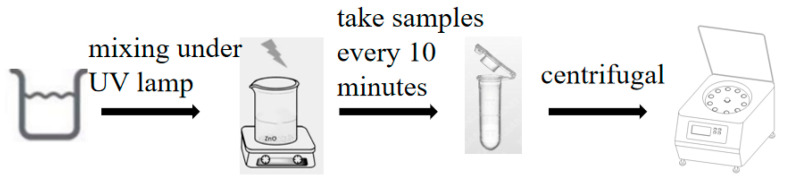
Photocatalytic degradation of dyes.

Figure 15 shows the photocatalytic degradation of the dye. In this experiment, a 250 W UV lamp was used as the light source, with the emission primarily centered at approximately 365 nm (near ultraviolet). Methylene blue (MB) was selected as the target compound for degradation. Prior to the experiment, 50 mg of the catalyst was evenly dispersed in 50 mL of a 10 mg/L MB solution, followed by 30 min of stirring in the dark to reach adsorption–desorption equilibrium. The UV lamp was then turned on, and samples were collected every 10 min. After centrifugation, the transparent supernatant was separated using a pipette. Finally, the absorption intensity of MB in each sample was measured using a UV-Vis spectrophotometer to evaluate the photocatalytic degradation efficiency. The optimal photocatalytic parameters were identified, and the effects of stirring and light source (sunlight vs. UV light) on the photocatalytic degradation efficiency of ZnO were further analyzed.

### 3.3. Catalyst Recycling

Based on the comparison of catalytic activity, the ZnO sample with optimal photocatalytic performance (synthesized at 300 °C for 1.5 h) was selected for the catalyst recycling experiments. The procedure is as follows: after each photocatalytic test, the sample was recovered by centrifugation, washed five times with deionized water, followed by five washes with anhydrous ethanol. It was then centrifuged, dried, and subjected to the next round of photocatalytic experiments to assess its photodegradation performance and stability.

## 4. Conclusions

This study synthesized rod-shaped ZnO nanoparticles through grinding and calcination, investigating the effect of various annealing temperatures and times on the photocatalytic activity of ZnO. The samples were characterized by techniques including XRD, FTIR, SEM, mapping, XPS, and UV-visible spectroscopy to analyze their composition, structure, and morphology. MB solution was chosen as the target pollutant to assess the photocatalytic performance, cycling stability, and degradation efficiency of ZnO under different annealing conditions. The results show that both annealing temperature and time significantly influence the morphology and photocatalytic properties of the materials. The photocatalytic degradation performance of the ZnO catalyst was evaluated under UV light, and the photocatalytic efficiency under different lighting conditions and stirring states followed the order: η _stirring under sunlight_ > η _sunlight_ > η _stirring under UV light_ > η _UV light_. The degradation efficiency of MB exceeded 98% after 40 min of exposure to both sunlight and UV light. The photocatalysts demonstrated excellent stability, as cycling tests under UV light revealed that catalytic efficiency remained above 90% after five cycles. This indicates that these photocatalysts possess outstanding photocatalytic activity and stability, showing promising potential for applications in the photocatalytic degradation of organic dyes.

## Figures and Tables

**Table 1:** Band gap width (Eg) of ZnO under different annealing conditions.

Terms	Eg/eV
250 °C 2 h	3.2122
300 °C 2 h	3.1882
350 °C 2 h	3.1689
400 °C 2 h	3.1681
300 °C 1 h	3.1812
300 °C 1.5 h	3.1603

**Table 2:** Degradation rate of ZnO, first-order reaction kinetic constants ka and R^2^ under different annealing condition.

Terms	Degradation Rate	ka/min^−1^	R^2^
250 °C 2 h	83.15%	0.05154	0.94598
300 °C 2 h	99.86%	0.10793	0.99034
350 °C 2 h	98.04%	0.02897	0.9974
400 °C 2 h	97.86%	0.0513	0.95658
300 °C 1 h	99.84%	0.11365	0.99521
300 °C 1.5 h	99.88%	0.12432	0.99501
UV light	90.15%	0.06051	0.94364
UV light + stirring	98.78%	0.1067	0.9403
sunlight	99.77%	0.12986	0.81995
sunlight + stirring	99.72%	0.13423	0.77258

**Table 3:** Degradation of MB in other investigations.

Sr. no.	Catalyst	Morphology	Catalyst Loading	Organic Pollutant	Light Source	Irradiation Time (min)	% Degradation	Reference
1	ZnO	Ouasi-spherical/ellipsoidal	0.1 g/L	10 mg/L	Solar	120	96.38	[59]
2	ZnO NPs	Stick-shaped	0.2 g/L	10 mg/L	Sunlight	150	94	[60]
3	ZnO NPs	Agglomerated	0.2 g/L	20 mg/L	Sunlight	180	91.4	[61]
4	ZnO NPs	Spherical	0.2 g/L	10 ppm	UV light	105	98	[62]
5	ZnO NPs	Spherical	1.0 g/L	50 ppm	Sunlight	90	96	[63]
6	ZnO NPs	Spherical	0.4 g/L	5 ppm	Sunlight	100	95.1	[64]
7	ZnO NPs	Spherical	0.8 g/L	50 μM	UV light	180	74	[65]
8	ZnO NPs	Spherical	1.0 g/L	10 μM	Sunlight	90	~100	[66]
9	ZnO NPs	Spherical	0.6 g/L	10 μM	Sunlight	50	98.17	[67]
10	ZnO NRs	Stick-shaped	0.6 g/L	10 μM	Sunlight	60	99	[68]
11	ZnO NPs	Flower-shaped	0.5 g/L	20 ppm	Sunlight	120	95	[69]
12	ZnO NPs	Stick-shaped	0.5 g/L	100 ppm	UV light	200	69	[70]
13	ZnO NPs	Semi-spherical	1.0 g/L	15 ppm	Hg lamp	90	92.78	[71]
14	ZnO NPs	Spherical	0.6 g/L	10 μM	Sunlight	90	94.07	[72]
15	ZnO NRs	Stick-shaped	1.0 g/L	10 mg/L	UV light	40	90.15	This work
16	ZnO NRs	Stick-shaped	1.0 g/L	10 mg/L	UV light + stirring	40	98.78	This work
17	ZnO NRs	Stick-shaped	1.0 g/L	10 mg/L	Sunlight	40	99.77	This work
18	ZnO NRs	Stick-shaped	1.0 g/L	10 mg/L	Sunlight +stirring	40	99.72	This work

## Data Availability

Data are contained within the article.
